# Estimating health plan costs with the OneHealth tool, Cambodia

**DOI:** 10.2471/BLT.17.203737

**Published:** 2018-06-04

**Authors:** Catherine Barker Cantelmo, Momoe Takeuchi, Karin Stenberg, Lo Veasnakiry, Ros Chhun Eang, Mo Mai, Eijiro Murakoshi

**Affiliations:** a Palladium, 1331 Pennsylvania Ave NW, Suite 600, Washington, DC 20002, United States of America.; b Country Office, World Health Organization, Hanoi, Viet Nam.; c Department of Health Systems Governance and Financing, World Health Organization, Geneva, Switzerland.; d Department of Planning and Health Information, Ministry of Health, Phnom Penh, Cambodia.; e Payment Certification Agency, Ministry of Health, Phnom Penh, Cambodia.; f Country Office, World Health Organization, Phnom Penh, Cambodia.; g Global Link Management Inc., Tokyo, Japan.

## Abstract

**Objective:**

To do resource and cost projections for the entire Cambodian health sector using the OneHealth tool, during the development of the third national health strategic plan 2016–2020.

**Methods:**

Through a consultative process, the health ministry estimated the needed and available resources to implement the strategic plan. The health ministry used the OneHealth Tool to estimate costs of expanding public sector service provision and compared these to estimates of projected available financing. Cost estimates covered implementation of health programmes including commodities and programme management costs, and six cross-cutting health system strengthening components. The tool is populated with local demographic, epidemiological, programmatic and unit cost data. We present costs in constant 2015 United States dollars (US$).

**Findings:**

We estimated the five-year cost of the strategic plan to be US$ 2973.8 million. Costs are split between health systems strengthening components (US$ 1516.3 million) and investments in individual disease or public health programmes (US$ 1457.5 million). Health programmes for maternal and neonatal health (US$ 367 million), child health and immunization (US$ 197 million) and noncommunicable disease (US$ 157 million) have the highest costs. Although projected resource needs increase over time, a financial space analysis with ambitious projected increases in government funding indicates that government and donor funding jointly could be sufficient to cover the cost of the strategic plan from 2018 to 2020.

**Conclusion:**

The results both informed development of the strategic plan, and contributed to the evidence base for improved budgeting, resource mobilization strategies and stronger overall public sector financial planning.

## Résumé

**Objectif:**

Effectuer des projections des ressources et des coûts pour l'ensemble du secteur de la santé au Cambodge à l'aide de l'outil OneHealth, dans le cadre de l'élaboration du troisième plan stratégique national de santé 2016-2020.

**Méthodes:**

Par le biais d'un processus consultatif, le ministère de la Santé a estimé les ressources nécessaires et disponibles pour mettre en œuvre le plan stratégique. Le ministère de la Santé a utilisé l'outil OneHealth pour estimer les coûts liés au développement de la fourniture de services du secteur public, puis comparé ces coûts aux estimations du financement disponible prévu. Les estimations de coûts couvraient la mise en œuvre de programmes de santé, et notamment les coûts des produits de base et de gestion des programmes, et six composantes transversales du renforcement du système de santé. L'outil est alimenté par des données locales relatives à la démographie, à l'épidémiologie, aux programmes et au coût unitaire. Nous présentons les coûts en dollars des États-Unis constants de 2015.

**Résultats:**

Selon nos estimations, le coût pour cinq ans du plan stratégique s'élève à 2973.8 millions de dollars des États-Unis. Les coûts sont divisés entre les composantes du renforcement des systèmes de santé (1516.3 millions de dollars des États-Unis) et les investissements dans différentes maladies ou des programmes de santé publique (1457.5 millions de dollars des États-Unis). Les programmes pour la santé de la mère et du nouveau-né (367 millions de dollars des États-Unis), la santé et la vaccination des enfants (197 millions de dollars des États-Unis) et les maladies non transmissibles (157 millions de dollars des États-Unis) représentent les coûts les plus élevés. Bien que les besoins projetés en ressources augmentent avec le temps, une analyse financière incluant des hausses prévues ambitieuses du financement gouvernemental indique que, pris ensemble, le financement gouvernemental et le financement des donateurs pourraient suffire à couvrir le coût du plan stratégique de 2018 à 2020.

**Conclusion:**

Les résultats ont servi de base à l'élaboration du plan stratégique et enrichi le corpus de données pour une meilleure budgétisation, des stratégies de mobilisation de ressources et un renforcement de la planification financière du secteur public général.****

## Resumen

**Objetivo:**

Realizar estimaciones de recursos y costes del sector de la salud de Camboya utilizando la herramienta OneHealth, durante el desarrollo del tercer plan estratégico nacional de salud 2016-2020.

**Métodos:**

A través de un proceso de consulta, el Ministerio de Salud estimó los recursos necesarios y disponibles para implementar el plan estratégico. El Ministerio de Salud utilizó la Herramienta OneHealth para estimar los costes de la ampliación de la prestación de servicios del sector público y los comparó con las estimaciones de la financiación prevista disponible. Las estimaciones de costes abarcaron la implementación de programas de salud, incluidos los gastos de gestión de productos básicos y programas, y seis componentes transversales de consolidación del sistema de salud. La herramienta cuenta con datos demográficos, epidemiológicos, programáticos y de costes unitarios locales. Presentamos los costes en dólares estadounidenses constantes de 2015 (USD).

**Resultados:**

Calculamos que el coste a cinco años del plan estratégico sería de 2973.8 millones de USD. Los costes se dividen entre los componentes de consolidación de los sistemas de salud (1516.3 millones de USD) y las inversiones en enfermedades específicas o en programas de salud pública (1457.5 millones de USD). Los programas de salud para la salud materna y neonatal (367 millones de USD), la salud e inmunización infantil (197 millones de USD) y las enfermedades no transmisibles (157 millones de USD) suponen los costes más altos. Aunque las necesidades de recursos estimados aumentan con el tiempo, un análisis de espacio financiero con aumentos ambiciosos previstos en la financiación del gobierno, indica que el financiamiento del gobierno y de los donantes en conjunto podría ser suficiente para cubrir el coste del plan estratégico de 2018 a 2020.

**Conclusión:**

Los resultados informaron sobre el desarrollo del plan estratégico y contribuyeron a la base empírica para un mejor presupuesto, estrategias de movilización de recursos y una planificación financiera general más sólida del sector público.

## ملخص

**الهدف:**

وضع تقديرات للموارد والتكاليف لقطاع الصحة في كمبوديا بالكامل، وذلك باستخدام أداة OneHealth، أثناء وضع الخطة الاستراتيجية الوطنية الثالثة للصحة خلال الفترة من 2016 إلى 2020.

**الطرق:**

من خلال عملية تشاورية، قدرت وزارة الصحة الموارد اللازمة والمتاحة لتنفيذ الخطة الاستراتيجية. استخدمت وزارة الصحة أداة OneHealth لتقدير تكاليف التوسع في توفير خدمات القطاع العام، ومقارنتها بتقديرات التمويل المتاح المتوقع. وغطت تقديرات التكاليف تنفيذ البرامج الصحية بما في ذلك تكاليف السلع وتكاليف إدارة البرنامج، وستة مكونات شاملة لتعزيز النظام الصحي. يتم تغذية الأداة بالبيانات الديموغرافية المحلية، والبيانات الخاصة بالأوبئة، والبيانات البرمجية وتكاليف الوحدات. نحن نقدم التكاليف بالقيمة الثابتة لدولار الولايات المتحدة الأمريكية لعام 2015 (الدولار الأمريكي).

**النتائج:**

كان تقديرنا لتكلفة الخطة الاستراتيجية لمدة خمس سنوات بقيمة تبلغ 2973.8 مليون دولار أمريكي. يتم تقسيم التكاليف بين مكونات تعزيز الأنظمة الصحية (1516.3 مليون دولار أمريكي)، والاستثمارات في البرامج الفردية للأمراض أو برامج الصحة العامة (1457.5 مليون دولار أمريكي). وكانت البرامج الصحية للأمهات والأطفال حديثي الولادة (367 مليون دولار أمريكي)، وصحة الأطفال وعمليات التمنيع (197 مليون دولار أمريكي)، وتكاليف الأمراض غير المعدية (157 مليون دولار أمريكي)، هي الأعلى من حيث التكاليف. على الرغم من أن الاحتياجات المتوقعة من الموارد تتزايد مع مرور الوقت، فإن التحليل المالي للوضع الاستراتيجي وتقييم الإجراءات، إلى جانب الزيادات المتوقعة الطموحة في التمويل الحكومي، تشير جميعها إلى أن التمويل من الحكومة والجهات المانحة معاً يمكن أن يكون كافياً لتغطية تكلفة الخطة الاستراتيجية من عام 2018 إلى عام 2020.

**الاستنتاج:**

كشفت النتائج عن تطوير في الخطة الاستراتيجية، كما ساهمت في قاعدة الأدلة على تحسين الميزانية، واستراتيجيات تعبئة الموارد، وكذلك التخطيط المالي الأقوى بشكل إجمالي للقطاع العام. ****

## 摘要

**目的:**

在 2016 至 2020 年第三个国家健康战略规划发展期间，通过使用同一健康 (OneHealth) 方法为整个柬埔寨卫生部门进行资源和成本预测。

**方法:**

通过协商过程，卫生部估算了实施该战略规划的必需和可用资源。卫生部使用同一健康 (OneHealth) 方法估算了扩大公共部门服务供应的成本并与预计可用财政预算进行比较。成本预算覆盖健康规划的实施，包括商品和规划管理成本以及六个交叉的健康系统强化组成部分。该工具多用于当地人口统计学、流行病学、程序和单位成本数据。我们以固定的 2015 年美元 (US$) 来展示成本费用。

**结果:**

我们估算实施五年战略规划的成本为 2973.8亿美元。成本分为健康系统强化组成部分（1516.3亿美元）以及个人疾病或公共健康规划投资（1457.5亿美元）。针对孕产妇和新生儿健康（367 亿美元）、儿童健康和免疫接种（197 亿美元）和非传染性疾病（1.57 亿美元）的健康规划成本最高。尽管预计所需资源会随着时间的推移不断增加，但一项对政府资金投入充满雄心壮志的预算增加财务空间分析表明，政府和捐助者的共同资金能够充分覆盖 2018 至 2020 年间的战略规划成本。

**结论:**

以上结果均通报了该战略规划的发展，并为改进预算、资源调动战略和更强大的整体公共部门财务规划提供了证据基础。****

## Резюме

**Цель:**

Составить прогнозы ресурсов и расходов для всего сектора здравоохранения Камбоджи, используя инструмент OneHealth, в ходе разработки третьего национального стратегического плана в области здравоохранения на 2016–2020 годы.

**Методы:**

В ходе совещания Министерство здравоохранения провело оценку необходимых и доступных ресурсов для реализации стратегического плана. Министерство здравоохранения использовало инструмент OneHealth для оценки расходов на расширение предоставления услуг в государственном секторе и сравнило их с оценками прогнозируемого финансирования. Оценка расходов охватывала реализацию программ в области здравоохранения, включая расходы на товары и услуги коммерческого характера и руководство программами, а также шесть межотраслевых компонентов укрепления системы здравоохранения. Инструмент учитывает местные демографические и эпидемиологические данные, расходы на реализацию программ, а также удельные расходы. Величина расходов представлена в постоянных ценах 2015 года в долларах США (долл. США).

**Результаты:**

Авторы подсчитали, что величина расходов на реализацию стратегического плана, рассчитанную на 5 лет, составит 2973.8 млн долл. США. Расходы распределены между компонентами укрепления систем здравоохранения (1516.3 млн долл. США) и инвестициями в программы по борьбе с отдельными заболеваниями или программы в области общественного здравоохранения (1457.5 млн долл. США). Наибольшие расходы связаны с программами по охране здоровья матерей и новорожденных (367 млн долл. США), охране здоровья детей и иммунизации (197 млн долл. США) и борьбе с неинфекционными заболеваниями (157 млн долл. США). Хотя прогнозируемые потребности в ресурсах с течением времени возрастают, анализ финансового пространства со значительным плановым увеличением государственного финансирования показывает, что совместного государственного и спонсорского финансирования может быть достаточно для покрытия расходов на реализацию стратегического плана с 2018 по 2020 год.

**Вывод:**

Полученные результаты послужили основой для разработки стратегического плана и способствовали созданию доказательной базы для совершенствования составления бюджета, стратегий мобилизации ресурсов и укрепления финансового планирования в государственном секторе.

## Introduction

During development of national health plans, governments need to understand the costs and resource implications of proposed strategies. Estimating resources required for implementation allows decision-makers to consider the feasibility and affordability of the plan. Cost projections guide allocations of scarce resources within the health sector. Such projections also inform decisions on resource allocation to the health sector in relation to other public and social sectors. Conducting cost analyses to inform development of national health plans is considered best practice. However, few publications, mostly grey literature, describe how assessments of health sector-wide resource needs inform national-level planning and decision-making on budget allocation.^1^^–^^6^

For countries to achieve the sustainable development goal (SDG) 3, that is, to “ensure healthy lives and promote well-being for all at all ages,” in pursuit of universal health coverage (UHC), decision-makers need to understand the related resource requirements. In Cambodia, the government developed the third national health strategic plan 2016–2020 to guide progress towards UHC. The strategic plan is built on two previous health sector plans, which focused on expanding availability and accessibility of key services to achieve the millennium development goals. The new plan reflects the vision, goals and targets of the SDGs, and aims to ensure that quality health services are geographically and financially accessible and equitably provided to all Cambodians.^7^

The strategic plan also addresses the financing challenges Cambodia is facing when moving towards UHC. The country implemented several health reforms over the last decade, including various contracting and incentive mechanisms and introduction of the health equity funds, a third party subsidy scheme for poor people.^8^ These reforms have increased equitable access to health-care services. Yet, out-of-pocket payments (OOP) are high and public spending on health is low (Sokkheang L, Ministry of Finance and Economy of Cambodia, 2016, unpublished data; Table 1).^7^^,^^9^^,^^10^

**Table 1:** Health financing indicators for the third national health strategic plan 2016–2020, Cambodia

Indicator	Baseline (2016)	Target (2020)
**Total health expenditure, US$ in million**	1207	NA
Expenditure from government, amount (%)	269 (22.3)	NA
Expenditure from OOP, amount (%)	729 (60.4)	NA^a^
Expenditure from external sources, amount (%)	200 (16.6)	NA
**Government health expenditures of GDP, %^b^**	1.2	2.0
**Households with catastrophic health care expenditure, %**	4.7	< 1.0
**Population covered by social health protection systems, %**	23.0	50.0

GDP: gross domestic product; NA: not applicable; OOP: out-of-pocket payments; US$: United States dollars.

^a^ Target set to be below 40%.

^b^ GDP in 2016 was projected to be US$ 20615 million and the government estimates the GDP to be US$ 29764 million in 2020.

Note: Expenditure from health insurance sector is not included.

Sources: National Health Accounts (NHA) 2016; The third health strategic plan 2016–2020.^9^^,^^10^

Total health expenditure in Cambodia increased from 1057 million United States dollars (US$) in 2014 to US$ 1207 million in 2016. While OOP increased from US$ 664 million in 2014 to US$ 729 million in 2016, the proportion of OOP of the total health expenditure declined slightly from 63.3% in 2014 to 60.4% in 2016. However, the government only spent 1.2% of its gross domestic product (GDP) on health expenditures, which is below the average for low- and middle-income countries in the Western Pacific Region (Sokkheang L, Ministry of Finance and Economy of Cambodia, 2016, unpublished data; Table 1).^9^

The health ministry plans to scale up coverage of key interventions from 2016 to 2020 (Table 2). While many interventions already have high coverages, the government plans to rapidly scale-up coverage of noncommunicable disease and mental health services, as coverage for these services remains low. To reduce OOP and offer improved financial protection, the government plans to expand its social health protection schemes during implementation of the strategic plan, to include additional vulnerable populations, such as people with disabilities, older people and children younger than five years. Under the strategic plan, the government targets to cover half of the entire population through health equity funds by 2020.

**Table 2:** Selected coverage targets within the third national health strategic plan 2016–2020, Cambodia

Indicator	Coverage target	
	2015	2020
Women using modern contraceptive methods, %	40	46
Pregnant women that receive four or more ANC consultations by health personnel, %	79	89
Adult population living with HIV that receive ART, %^a^	79	94
Women aged 30–49 years screened for cervical cancer at least once, %	5	10
Adults older than 39 years screened for cardiovascular disease or diabetes, %	6	18

ANC: antenatal care; ART: antiretroviral treatment; HIV: human immunodeficiency virus.

^a^ Adult population represents people older than 14 years.

A prerequisite for ensuring adequate funding to meet health impact targets and improve financial risk protection is estimating the resources needed for implementation of the plan. The government outlined the specific strategies to be implemented from 2016 to 2020 and estimated the associated resource requirements; this was the first instance of the government conducting a resource needs projection for a multi-year strategy covering the entire health sector.

To estimate resource requirements for the strategic plan, the Cambodian health ministry selected the OneHealth tool,^11^ a tool developed to inform national strategic health planning (Box 1). Here we describe how the health ministry used the tool to inform development and prioritization of the strategy and its targets, how much it will cost to implement the plan, and if there are sufficient financial resources available to cover costs.

Box 1 **Overview of the OneHealth tool^11^**

**What is the OneHealth tool?**

The tool is developed to forecast the costs and health impacts associated with investments in the health system, thereby informing medium- to long-term strategic planning. Since its inception in 2009, the development and application of the tool is overseen by a United Nations inter-agency working group.

**What types of costs are estimated in the tool, and how?**

The tool estimates the costs of service delivery under individual health programmes and the costs of cross-cutting health system components, including infrastructure, human resources for health, logistics, health information systems, health financing and governance. The tool uses a bottom-up, ingredients-based costing approach, whereby standards and guidelines for quality clinical care are translated into quantities of inputs required per year. Quantities are multiplied by input-specific prices which can be set up to change over time.

**What are other features of the tool?**

The tool is integrated in the Spectrum suite of software models, which allows for the linking of investment targets to various health outcome and impact models, such as the Lives Saved Tool. The link to epidemiological models allows for health services to be dynamically estimated over time, taking into account population growth, reduced mortality and reduced incidence or prevalence of disorders as coverage of interventions (preventive and curative) increases. The OneHealth Tool adds value to other existing tools since it links different disease projection models, and links the service targets to health system requirements. The tool can be used to model scenarios around different delivery strategies and targets and to assess associated cost and impact.

**What are some of the tool inputs?**

- Epidemiological data (e.g. prevalence or incidence of particular diseases or conditions);
- Baseline and targeted intervention coverage;
- Health programme activity requirements (e.g. health personnel training, mass media campaigns);
- Health system requirements and targets (e.g. planned numbers of health workers to be employed or health facilities to be constructed and equipped);
- Prices of commodities and other inputs.

The tool is pre-populated with data from global literature and country-specific health and population surveys; however the user can change input assumptions when more appropriate local data is available.

**What are some of the tool cost outputs?**

- Annual and total costs by health programme, health system component or type of cost;
- Health programme and health system requirements (e.g. number of health workers needed to meet intervention coverage targets)

**Where has the tool been applied?**

In several low-and middle-income countries to inform health sector planning processes and has also been used to generate estimates for global advocacy efforts.^2^^–^^6^^,^^12^^–^^16^

## Methods

**Fig. 1:**
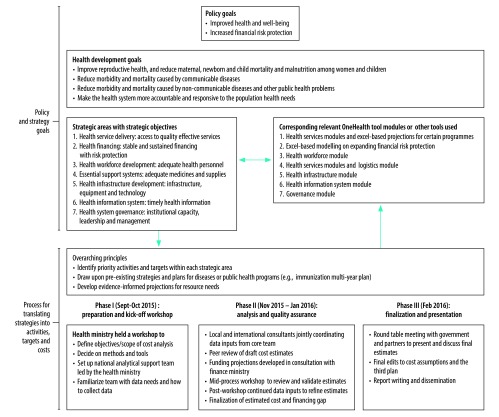
Priorities and development process of Cambodia’s third national health strategic plan 2016–2020

The health ministry began strategic plan development in 2015 using an inclusive bottom-up approach (Fig. 1). A core team of health ministry officials and development partners guided the process, under which six task teams were created for service delivery; financing; health workforce; health information; physical infrastructure; and essential support services and governance. The task teams reviewed achievements and progress made under the previous plan and identified challenges, opportunities, priorities and policy recommendations for achieving UHC.

### Scale-up strategies

The government plans to increase the number of health centres in Cambodia from 1183 in 2016 to 1351 in 2020 to expand access to services. The projections also indicate that Cambodia will meet facility staffing targets by 2020, as set by the national Health Workforce Development Plan 2016–2020.^17^ In this period, the government aims to increase the total number of public sector health workers from 25 294 to 37 925, with the biggest increases seen among secondary nurses, medical doctors and pharmacists. Meeting these targets will lead to an estimated 7.8 nurses, 2.8 midwives and 2.4 doctors per 10 000 population in the public sector.

### Projecting resource requirements

To estimate costs of expanding public sector service provision, the health ministry chose the OneHealth tool, which incorporates many other disease or programme-specific tools used previously in Cambodia.^12^ The tool is a freely available software platform, whose development is overseen by the World Health Organization (WHO) and other agencies.^11^ Information on the methods for calculating costs is summarized in Box 1 and available in detail elsewhere.^2^^–^^6^^,^^11^^,^^13^ Briefly, the tool is populated with country data on epidemiology, demography, treatment guidelines for effective interventions and health system planning modules, which allows users to project resource needs based on population health needs and strategic targets.

The health ministry, development partners and other stakeholders attended tool training and data validation workshops and a high-level consultation meeting to discuss investment strategies, targets, cost scenarios and funding gaps. The health ministry formed the OneHealth tool costing team, who used the tool to estimate approximately 74% of total projected costs of the strategic plan; they calculated the remaining costs using Excel spreadsheets (Microsoft, Redmond, United States of America; Fig. 1). The health ministry designed two scenarios for discussion due to uncertainty in the percentage of people who will access services in the public versus private sector. The first scenario assumed that the proportion of total health services delivered in the public sector remains constant. The second assumed that the quality of public sector service delivery would improve under the strategic plan, resulting in a 25% increase in public versus private sector health service utilization. The health ministry selected the second scenario for inclusion in the new strategic plan due to planned quality improvements.

The OneHealth Tool costing team comprised of four groups: communicable disease; noncommunicable diseases; reproductive, maternal, neonatal, and child health; and health systems. Data from subsector strategic plans, (e.g. Strategic Plan for HIV/AIDS [human immunodeficiency virus/ acquired immune deficiency syndrome] and STI [sexual transmitted infections] Prevention and Control in the Health Sector 2016–2020), the health management information system, demographic and health surveys, and other health and disease-burden studies informed the development of assumptions on disease burden and population coverage in the tool.^7^^,^^12^^,^^17^^–^^19^ Health ministry staff and other public sector stakeholders, such as the central medical store, reviewed and provided cost assumptions. When Cambodia-specific data were unavailable, we used default regional averages available in the tool for South-East Asia or entered estimates of expert opinion into the tool. We present costs in constant 2015 US$. Box 2 shows the types of costs estimated, including commodity and programme management costs to implement health programmes grouped into nine categories and costs of six cross-cutting health systems strengthening components.

Box 2 **Types of costs included in cost projections for the third Health Strategic Plan 2016–2020, Cambodia**

**Cross-cutting health systems costs**

Human resource for health: total remuneration for staff, education and training, and administrative costs;

Logistics: central medical store operating costs for storage and transportation, and cost of drugs and supplies which are procured but not consumed (wastage);

Infrastructure: construction of new facilities, add-ons to existing facilities, facility operating costs, procurement and maintenance of facility equipment;

Health information systems: cost of operating health management and information systems, including information and communication technology,

Health financing: administration and benefit transfer costs for the health equity funds;

Governance: costs of laboratory quality management, private sector regulation, hospital quality assurance and management and infection control and prevention.

**Health programme costs**

Cost of medications, supplies and programme management for programmes grouped into nine categories: (i) reproductive, maternal, and newborn health; (ii) noncommunicable diseases; (iii) mental health; (iv) child health and immunization; (v) malaria and dengue; (vi) tuberculosis; (vii) HIV; (viii) nutrition; (ix) all other programs (eye health, promotive health, leprosy and infectious disease control).

HIV: human immunodeficiency virus.

### Estimating financial space

The health ministry estimated the financial resources that could be mobilized from the government, development partners and households between 2016 and 2020. Government health contributions were modelled based on the economy and finance ministry’s predictions of a 7% annual GDP growth and meeting the health ministry’s target of increasing government health expenditure from 1.3% to 2% of GDP from 2016 to 2018 (Sokkheang L. Ministry of Finance and Economy of Cambodia, 2016, unpublished data). For this analysis, we assumed a constant government health spending rate of 2% of GDP from 2018 to 2020. We modelled external resources using WHO projections for donor funding up to 2020. These projections are based on unofficial discussions between WHO and donors. We based private expenditure projections on regression analysis of total health expenditure, minus the estimated government and external expenditure. Due to uncertainty in factors that influence OOP, such as the cost of user fees, the willingness and ability of people to pay these fees and expansion of financial protection schemes for health, we did not estimate OOP as a proportion of private health expenditure.

## Results

### Resource requirements

We estimate that the five-year cost of the strategic plan will be US$ 2974 million, with annual costs increasing from US$ 537 million in 2016 to US$ 668 million in 2020. Estimations suggest that resource needs for public health sector will increase from US$ 32 to US$ 38 per capita during this period. However, assuming 7% GDP growth annually, public health sector costs as a percentage of nominal GDP would decline from 2.6% (US$ 537 million/US$ 20615 million) in 2016 to 2.2% (US$ 668 million/US$ 29764 million) in 2020.

#### Health programmes

**Fig. 2:**
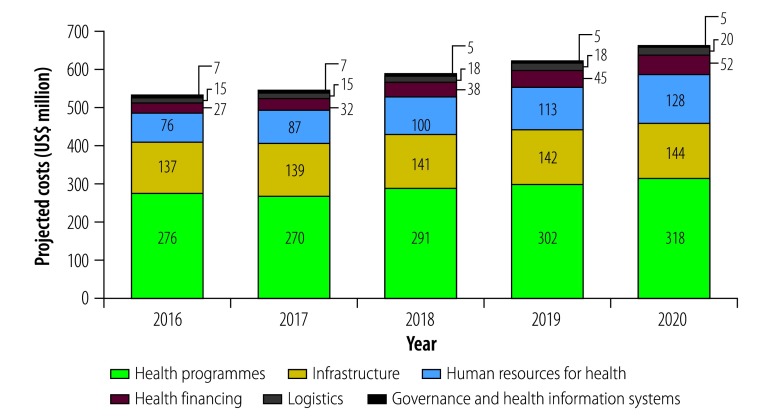
Projected annual health systems costs of the third national health strategic plan 2016–2020, by cost area, Cambodia

About half (US$ 1457 million) of the total five-year costs are for specific investments related to individual diseases or public health programmes (Fig. 2). Commodity costs represent 41% (US$ 596 million) of the total health programme costs and will increase 1.6 times during the period, due to increases in the projected number of services provided over time. Programme management costs, which include the cost of programme-specific training, monitoring and evaluation and other activities, will decrease over time from US$ 184 million to US$ 170 million, reflecting investments in support activities at the onset of the plan.

US$: United States dollars.

**Fig. 3:**
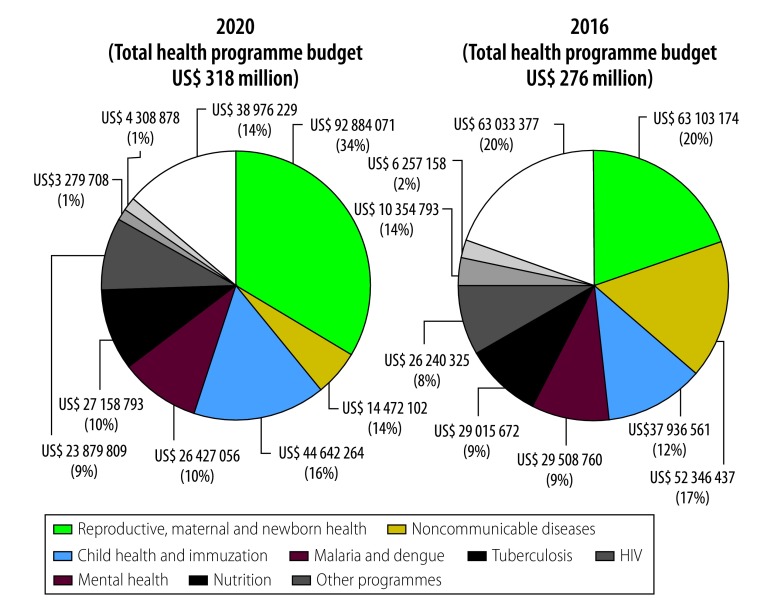
Projected health programme costs in 2016 versus 2020, Cambodia

HIV: human immunodeficiency virus; US$: United States dollars.

Note: Other programmes include eye health, promotive health, leprosy and infectious disease control.

The largest five-year costs for health programmes are for maternal and neonatal health (25%; US$ 366 million), child health and immunization (14%; US$ 197 million) and noncommunicable diseases (11%; US$ 157 million). If the service delivery targets for the public sector are achieved, the mental health and noncommunicable disease programmes will have the fastest projected growth in the share of overall costs (Fig. 3). The anticipated growth in these programmes’ costs is a result of scaling up coverage from current low levels. However, the analysis does not consider the costs or effects of introducing or scaling up noncommunicable disease preventive programmes, such as tobacco cessation policies and behaviour change programmes. While such interventions would reduce noncommunicable disease risk factors over time, the effects would not be seen in the short-term. The cost projections therefore conservatively assume constant noncommunicable disease prevalence until 2020.

#### Health systems

Just over half (US$ 1516 million) of the five-year costs are for health systems strengthening components. The costs are set to increase by 34%, from US$ 261 million in 2016 to US$ 350 million in 2020 (Fig. 2). The largest projected cost is for infrastructure, increasing from US$ 137 million in 2016 to US$ 144 million in 2020. The majority of infrastructure costs (US$ 443 million of US$ 703 million) are operational facility costs; nearly one-third (US$ 204 million) is for medical equipment and new construction accounts for just 7% (US$ 49 million). The human resource component constitutes the second-highest health systems cost (US$ 504 million). Three-quarters of these costs (US$ 378 million) are for health workers’ salaries, which are projected to increase over time due to planned increases in the number of health workers and 5% annual salary increases. The remaining cost is for pre-service training, central in-service training and support activities, including incentives for rural postings. Health workforce costs are relatively low (US$ 128 million of the US$ 668 million total cost in 2020, or 19% of resource needs estimates in 2020), which may be a result of low remuneration of health staff in the public sector. Nonetheless, the health ministry and other stakeholders validated all targets and assumptions. Administrative and benefits costs for health equity funds (excluding costs accounted for under individual disease programmes) will nearly double from 2016 to 2020 due to the planned expansion of health equity funds coverage.

### Financial space and funding gap

Between 2016 and 2020, we estimate that total health expenditure will increase from US$ 1365 million to US$ 2013 million. This estimation is based on historical growth trends. Between 2007 and 2015, total health expenditure in Cambodia doubled. Estimation of the government proportion of projected total health expenditure shows an increase from 20% (US$ 274 million) in 2016 to 30% (US$ 595 million) in 2020. This assumption is ambitious, but the government has a strong commitment to health.^20^ We estimated that private expenditure as a proportion of the resources available would decrease slightly from 66% to 62% in the same timeframe, although such expenditure is projected to increase in absolute amounts, from US$ 943 million to US$ 1291 million. Donor contributions represent the smallest proportion (14%, US$ 147 million) of total health expenditure in 2016, and projected declines in donor support would result in donors representing approximately 8% (US$ 126 million) of total health expenditure in 2020.

**Fig. 4:**
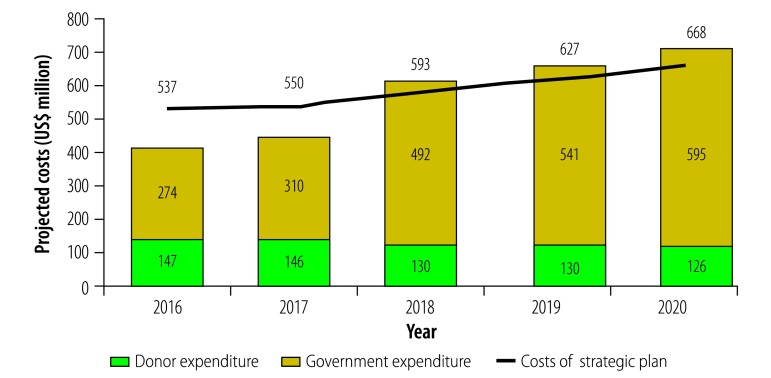
Projected third national health strategic plan 2016–2020 costs versus projected government and donor health expenditure, Cambodia

Cambodia could potentially mobilize sufficient funds from government and donor sources for implementation of the plan from 2018 and onward (Fig. 4). However, a funding gap would still exist at the onset of the plan, which may be filled by private sources (e.g. prepayment through formal sector and private insurance or OOP) if individuals are willing to pay for these health services.

## Discussion

Estimating costs and available financial space for the strategic plan has strengthened the health sector planning process in Cambodia, particularly for programmes that had not undergone a resource needs planning exercise before. For example, at the beginning of this exercise, the noncommunicable disease programme lacked a costed strategic plan and uncertainties existed about the expansion plan for the package of essential noncommunicable disease interventions. Through the costing process, the noncommunicable disease programme developed multiple cost scenarios for scaling up preventive and curative interventions. Based on these results, the decision-makers decided to focus on high-risk populations for cervical cancer and cardiovascular risk screening to contain costs, while expanding health education and preventive approaches for those at lower risk. The resource projections thus informed priority setting and the development of feasible targets by identifying health system and financial constraints in expanding access to services.

Simultaneously, the costing process justified ambitious scale-up plans for particular programmes in line with health needs and national priorities. The decision-makers for the tuberculosis programme opted for an ambitious quality improvement scenario, as this was deemed feasible given financial contributions from the Global Fund to Fight AIDS, Tuberculosis and Malaria.

The OneHealth tool was particularly useful for understanding health system requirements, which we found to be either lacking from costing exercises for vertical disease programmes or could have been double-counted when estimated separately by programme and then summed across programmes. The estimations also showed the gap between the current resource allocation and the resource requirements for different programme areas. The health ministry will use this information to discuss financing options, including resource mobilization strategies and to improve alignment of resource allocation based on the health needs of the population.

The financial space analysis demonstrated the need for a more detailed analysis of resource allocation and funding gaps for each health programme. In 2017, a WHO initiative collected information from development partners regarding pledged or expected financial contributions and technical assistance. This information will enable the health ministry to map funding projections of development partners to programmes and subprogrammes and can be used in health budget preparations and submission to the economy and finance ministry.

Linking national projections to subnational budget and planning processes may maximize the benefits of projections, as one of the weaknesses of the current programme-based budgeting process is that allocation to the subnational level is still based on historical expenditure, rather than resource-needs projections. Development partners including WHO are using results from the OneHealth tool to support a possible revision of programme-based budgeting structure and development of a formula or criteria for better subnational allocation.

The detailed cost estimates of health programmes, supplemented by the financial gap analysis, will inform health financing strategies as eligibility to receive external funding for health is reduced, especially for HIV/AIDS and tuberculosis. As Cambodia has experienced steady economic growth, various donors have started to reduce or withdraw their health sector funding support. Global health initiatives, such as the Global Fund or Gavi, the vaccine alliance, have requested increased government co-financing. Accordingly, the government has recently increased its budget contribution to cover health equity funds costs and procurement of antiretroviral, contraceptive and first-line tuberculosis medications. To plan for donor funding transitions and ensure sustainable financing of key health programmes, designing midterm funding plans and co-finance approaches to increase domestic funding and improve allocative and technical efficiency in the public health sector will be essential. The health ministry can use the cost analysis presented here for evidence-based transitional financing decisions.

Our analysis had several limitations. For most medications and supplies, Cambodia-specific prices were unknown. As a result, we adjusted default prices in the tool based on the average difference in costs for those commodities for which Cambodia-specific price data were available. A challenge was capturing costs for quality improvement measures, since explicit strategies had not yet been developed at the time of the analysis. Such costs should be considered in mid-term review and related updates of implementation costs. In terms of the financial space analysis, government projections were ambitious and OOP were not explicitly modelled.

The current analysis did not include health impact analyses in terms of progress towards the SDGs. The OneHealth tool includes a comprehensive set of impact models that have been used in other countries to analyse potential gains in averted mortality and morbidity, and includes outputs for many of the SDG health targets.^4^ Such analysis could help further inform decision-making in Cambodia.

Cost projections should be interpreted as reflective of a dynamic and uncertain context, thus necessitating updates of the projections over time. The Cambodian health ministry is moving to institutionalize processes that use evidence-based resource projections. While individual programmes have supported such processes, recent analysis has extended to include projections related to health equity funds expansion.^21^ The move towards modelling sector-wide costs for the strategic plan is an additional step in this direction, and the results strengthen annual budget development and negotiations with the economy and finance ministry. Institutionalization requires further investment in health ministry capacity to ensure that regular updates of resource projections are made during multi-year operational planning or during the mid-term review of the strategic plan. The costing of the plan has already led to further analysis; next steps may be to expand such analyses to fully identify funding and implementation gaps that can inform both annual budget planning processes as well as the future medium-term expenditure framework.
