# Corrigendum

**DOI:** 10.2471/BLT.18.101018

**Published:** 2018-10-01

In: Cantelmo CB, Takeuchi M, Stenberg K, Veasnakiry L, Eang RC, Mai M, et al. Estimating health plan costs with the OneHealth tool, Cambodia. Bull World Health Organ. 2018 July 1;96(7):462–70. http://dx.doi.org/10.2471/BLT.17.203737

on page 466, Fig. 3, should be as follows: 

**Fig. 3:**
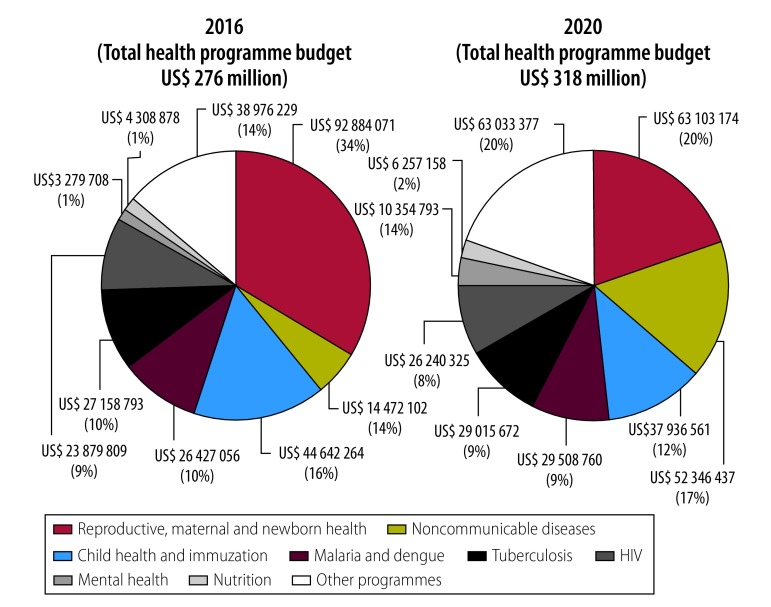
Projected health programme costs in 2016 versus 2020, Cambodia

HIV: human immunodeficiency virus; US$: United States dollars.

Note: Other programmes include eye health, promotive health, leprosy and infectious disease control.

